# Association between time to treatment and financial toxicity among women with breast cancer in Ethiopia: a multicentre study in a low-resource setting

**DOI:** 10.1007/s00520-026-10570-9

**Published:** 2026-03-13

**Authors:** Anteneh Ayelign Kibret, Heng Jiang, Edom Seife Woldetsadik, Miliyard Demeke Tafese, Biniyam Tefera Deressa, Chaojie Liu

**Affiliations:** 1 School of Psychology and Public Health, La Trobe University, Bundoora, VIC Australia; 2 Department of Human Anatomy, School of Medicine, College of Medicine and Health Sciences, University of Gondar, Gondar, Ethiopia; 3 School of Population and Global Health, University of Melbourne, Melbourne, VIC Australia; 4 College of Health Sciences, Department of Oncology, Addis Ababa University, Addis Ababa, Ethiopia; 5 Adama Hospital Medical College, Adama, Ethiopia; 6 Jimma Oncology Centre, Jimma University, Jimma, Ethiopia

**Keywords:** Breast cancer, Financial toxicity, Treatment delay, Ethiopia

## Abstract

**Purpose:**

Breast cancer imposes a substantial financial burden on patients, especially in low-income countries like Ethiopia where out-of-pocket (OOP) payments dominate health financing. This study aimed to assess the association between time to treatment initiation and financial toxicity (FT), among women with breast cancer in Ethiopia.

**Methods:**

A cross-sectional study was conducted from July to September 2024 among 458 women receiving breast cancer care at three tertiary hospitals in Ethiopia. Participants were interviewed using a validated Amharic version of the COST-FACIT tool, with additional data obtained from interviews and clinical records. The primary outcome was FT score, which ranged from 0 to 44, with a lower score indicating greater financial toxicity. Multivariable linear regression was used to assess the association between FT and the total treatment interval (TTI) from symptom recognition to treatment initiation, adjusting for sociodemographic, clinical, health insurance, and cost-coping behavioral factors**.**

**Results:**

The median FT score was 10 (IQR:5 to 16), indicating a high level of financial toxicity among the study participants. Most participants (71%) had a TTI longer than 90 days, which was associated with higher financial toxicity (median FT score 9 compared with 14 in those with ≤ 90 TTI days, p < 0.001). Longer TTI was a significant predictor (*B* = –2.50; 95% CI: –4.06 to –0.95; p = 0.002) of higher financial toxicity, after adjustment for variations of covariates. Other significant predictors of higher financial toxicity included younger age (< 40 years: *B* = –4.37; p = 0.001; 40–59 years: *B* = –3.53; p = 0.003), lower income (≤ 2,100 ETB: *B* = –5.40; 2,101–3,254 ETB: *B* = –3.91; p < 0.001), rural residence (*B* = –2.28; p = 0.039), on active treatment (*B* = –3.59; p = 0.001), and reliance on borrowed/assisted financing for OOP (*B* = –4.59; p < 0.001). There also existed FT disparities across the three hospitals.

**Conclusion:**

Financial toxicity is evident in Ethiopian women with breast cancer, particularly among those with lower socioeconomic status. Longer TTI is associated with higher financial toxicity. These findings highlight the limited role of the current health insurance arrangement in addressing the level and inequality of financial toxicity associated with breast cancer care in Ethiopia.

**Supplementary Information:**

The online version contains supplementary material available at 10.1007/s00520-026-10570-9.

## Background

Cancer is among the most costly and resource-intensive medical conditions, and the financial burden of treatment is a growing global concern [1]. Breast cancer contributes substantially to this burden because care often involves multimodal treatment (e.g., surgery, radiotherapy, systemic and/or hormonal therapy) and long-term follow-up [2–5]. In Ethiopia, where financial protection for cancer care is limited, a survey of patients attending major hospitals in Addis Ababa reported an average annual medical expenditure of approximately USD 2,325 per breast cancer patient, alongside coping strategies such as asset sales and borrowing to pay for care [6].

In low-resource settings like Ethiopia, the financial impact of breast cancer extends beyond direct medical costs. Limited service availability means patients often travel long distances, incurring transport, accommodation, and food expenses [7]. Treatment can also disrupt employment for patients and caregivers, reducing household income and productivity and forcing families to use savings, borrow, sell assets, or cut spending on essentials such as food and education [6, 8]. These pressures can lead to severe financial hardship for households [9–11].

Financial hardship has important consequences, including reduced quality of life, emotional distress, treatment interruption, and poorer outcomes [12–15]. Timely initiation of treatment is essential for effective cancer care, yet delays remain common [16, 17]. Delays may increase financial hardship through repeated health system visits, advanced-stage presentation requiring more intensive care, and prolonged income disruption; conversely, financial hardship may itself delay care by limiting the ability to pay for diagnostics, transport, and treatment-related costs [18–21].

Despite these plausible pathways, there is a scarcity of studies exploring the association between delay in breast cancer care and financial hardship. Understanding the link between timeliness of care and financial hardship is essential for developing responsive cancer policies, designing patient support programs, and improving the affordability and equity of breast cancer care. Achieving the WHO’s target that 80% of women with breast cancer complete affordable multimodal treatment requires health systems to address both clinical and financial barriers [22]. In line with the United Nations Sustainable Development Goal 10 (Reduced Inequality), ensuring timely and affordable cancer care is critical to guaranteeing equal access to health services [23]. This study therefore assesses financial hardship among women receiving breast cancer care at tertiary hospitals in Ethiopia and examines its association with the time interval from symptom recognition to treatment initiation. By quantifying this relationship, the study contributes to a growing body of literature on cancer-related financial hardship, informing policies aimed at improving both affordability and timely access to care and provides context-specific evidence to inform national cancer control strategies.

## Methods

### Study design and population

We conducted a cross-sectional study among women diagnosed with breast cancer at three tertiary hospitals in Ethiopia between July and September 2024. Participants were women aged 18 years or older with histologically confirmed breast cancer who had initiated treatment and received at least one modality of care (surgery, chemotherapy, or radiotherapy), regardless of whether they had completed their entire treatment course at the time of the survey.

### Study setting

The study was conducted in Ethiopia, the second-most populous African nation, with a population of more than 110 million and GDP per capita USD 1,272 in 2023 [24].

According to the 8th National Health Account (NHA), Ethiopia’s total health expenditure rose from ETB 72 billion (USD 3.1 billion) in 2016–17 to ETB 127 billion (USD 3.62 billion) in 2019–20, representing 4.7% and 6.3% of country’s GDP, respectively. Per capita health expenditure also increased steadily over the past two decades, from USD 4.5 in 1995–96 to USD 33.2 in 2016–17 and USD 36.3 in 2019–20. Based on the 2021–22 Ethiopia Service Provision Assessment (SPA), which surveyed 1,158 health facilities nationwide, only 28% reported providing any cancer services [25]. Additionally, only three tertiary hospitals provide radiotherapy.

The study was conducted in three major public hospitals in Ethiopia that provide comprehensive breast cancer services, including radiotherapy, Black Lion Hospital (Addis Ababa), Jimma University Medical Centre (Jimma), and Hiwot Fana Comprehensive Specialized Hospital (Harar).

### Data collection tools and procedures

#### Outcome variable

Financial toxicity was the outcome variable, a concept developed by Zafar and Abernethy [26]. Financial toxicity was defined as the distress or hardship patients suffering from the financial burden of cancer treatment [27].

We used the Comprehensive Score for Financial Toxicity (COST – FACIT) tool to measure financial toxicity. The COST—FACIT (Version 2) is designed to measure the FT of patients aged 18 years and older. It was developed by De Souza et al. and was validated to assess the degree of financial stress experienced by patients with cancer [28]. The COST questionnaire has 12 items that have been officially coded sequentially from FT1 to FT12. The responses vary from 0 (not at all), 1 (a little bit), 2 (somewhat), 3 (quite a bit), and 4 (very much). Items 2, 3, 4, 5, 8, 9, and 10 of the questionnaires are reverse scored. The scores of each item are cumulatively added to get the total score (0–44), with a lower score indicating a higher FT.

Formal permission letter from the COST-FACIT team and ethical approval from La Trobe University was obtained for developing the Amharic version of the COST-FACIT (supplementary file 1) through iterative forward–backward translation sequences. We followed the COST-FACIT procedure to translate and culturally adapt the tool, the steps include forward translation: The English COST-FACIT questionnaire was translated into Amharic languages, as appropriate, as possible to the original English COST-FACIT (Version 2) by two translators, who were fluent in the Amharic and English languages. The second step was reconciliation: the two forward translations were reconciled by a third translator, who was fluent in both Amharic and English languages. The third was back translation: the reconciled version of the questionnaire was then back translated to English by another translator, fluent in English and the native language. A FACIT staff member reviewed the back-translated items to identify any inaccuracies or inconsistencies from the forward translation and reconciliation process. A few comments raised by the FACIT representative were resolved. Once all issues were resolved and approved, the questionnaire was formatted by FACIT team and returned for proofreading. Subsequently, the cognitive debriefing script was translated and submitted to prepare for patient interviews.

To assess the feasibility and reliability of the translated tool in the Ethiopian context, a pilot study was conducted among 11 adult cancer patients at University Gondar specialized Comprehensive hospital to test for content validity. Participants completed the questionnaire independently, with interviewer assistance provided upon request. After completion, retrospective cognitive debriefing interviews were conducted by the data collector to assess comprehension, acceptability, and cultural appropriateness of the items. Participants were asked to identify any items they found confusing, irrelevant, or difficult to interpret. Feedback from these interviews was used to confirm the clarity of the tool. The pilot data demonstrated good internal consistency, with a Cronbach’s alpha of 0.75, supporting the reliability of the Amharic version of the COST–FACIT for use in this population. The FACIT approved validated Amharic version is now publicly accessible through the FACIT platform.

#### Exposure variable

Although the COST–FACIT tool asked respondents to rate their perceptions on financial toxicity within the prior seven days, these perceptions resulted from the accumulated financial burden of the respondents. We chose to examine the overall interval from symptom recognition to treatment initiation as our main exposure to capture the cumulative burden in a way that reflects patients’ lived experiences.

Total treatment interval (TTI) was defined as the time in days from the date of first symptom recognition to the date of initiation of cancer-specific treatment. This interval was calculated based on patient self-report and verified using medical records where possible.

#### Covariates

Potential covariates were identified based on existing literature. Studies have shown that financial toxicity is associated with many factors, including those patient-related (e.g. younger age, unemployment, unmarried status, low income) [29–32], cancer-related (e.g. advanced stage at diagnosis, receipt of adjuvant therapies, and multiple hospital admissions) [29], and health system-related (e.g. lack of health insurance, and greater distance from treatment centres) [29–32].

This study included sociodemographic, clinical, and health system–related variables known to influence financial toxicity.

Sociodemographic covariates included age at diagnosis (< 40, 40–59, ≥ 60 years), marital status (married/unmarried), residence (urban/rural), study site (Jimma, Black Lion, Hiwot Fana), per-capita household income tertiles (≤ 2,100; 2,101–3,254; ≥ 3,255 ETB), educational attainment (no formal education, primary/secondary, higher education), occupational status (employed/unemployed), family size (< 2, 2–5, > 6 members), use of traditional healing (yes/no), distance to the first healthcare facility (< 5 km vs ≥ 5 km), and health insurance status (yes/no). Distance to the healthcare facility refers to the distance from the participant’s residence to the first formal healthcare facility visited for the breast-related symptom.

Clinical covariates included medically confirmed comorbidity (yes/no), cancer stage (I–II vs III–IV), treatment modality (single, two, or three modalities), hospitalisations in the past year (0–3 vs > 3), and days since treatment initiation (≤ 180, 181–365, > 365 days). Treatment modality was categorised into three groups based on treatment intensity: single modality (surgery, chemotherapy, or radiotherapy only), two-modality combinations (surgery + chemotherapy, surgery + radiotherapy, or chemotherapy + radiotherapy), and all three modalities (patients who received surgery, chemotherapy, and radiotherapy). Days since treatment initiation was used as a proxy measure to describe participants’ position along the breast cancer care continuum,, informed by the National Comprehensive Cancer Network (NCCN) Guidelines for Survivorship [33]. Based on clinical milestones along the breast cancer care continuum, participants were initially classified into four groups: just started treatment (≤ 14 days since initiation), early–mid treatment (15–180 days), late treatment (181–365 days), and post-treatment (> 365 days). These categories were used for descriptive analyses to characterise the sample across clinically meaningful phases of care. For multivariable modelling, broader categories (≤ 180, 181–365, and > 365 days) were used to adjust for potential variation in financial toxicity related to timing of cost accumulation or recovery and three broader categories for multivariable modelling.

Variables measuring cost-related coping strategies included primary source of out-of-pocket payment (self-financed vs borrowed/assisted) and avoidance of care due to cost (yes/no).

#### Data collection and procedure

Data for this study were collected using an interviewer-administered structured questionnaire and by reviewing patients’ medical records. Information on key dates in the healthcare-seeking pathway was taken from hospital records and patient-held clinical files. To reduce recall bias, during interviews, participants were asked to link the timing of first symptom recognition and first healthcare visit to familiar calendar events, such as religious or national holidays, New Year celebrations, and school holidays. When participants could not remember the exact date, simple rules were used: the 15th of the month was recorded if only the month was remembered, the 5th if the event occurred at the beginning of the month, and the 25th if it occurred toward the end of the month. All eligible breast cancer patients presenting at the study sites during the data collection period were invited to participate. Eligibility was determined based on predefined inclusion criteria, and written informed consent was obtained prior to enrolment.

Face-to-face interviews were conducted by three trained female medical doctors who were not involved in the clinical care of participants. Interviews took place in private rooms to ensure confidentiality. All interviews were conducted in Amharic language. Each interviewer collected 65 to 252 questionnaires over the period from July and September 2024 and entered data into Redcap in English. To ensure data quality, the first author maintained daily communication with data collectors and conducted weekly site visits throughout the data collection period. All 458 returned questionnaires were complete and valid for analysis, as data collectors reviewed each form in real time to ensure no missing responses.

### Statistical analysis

Descriptive statistics were used to summarize patient characteristics, time intervals, and FT scores. Continuous variables were reported as means (± SD) or medians (IQR), while categorical variables were reported as frequencies and percentages.

Group differences in FT scores across categorical variables (e.g., age group, residence, education, health insurance status) were evaluated using non-parametric tests: Wilcoxon rank-sum test and the Kruskal–Wallis H test. Pearson’s correlation analysis was used to assess the strength and direction of the linear relationship between financial toxicity and time to treatment initiation.

Based on the WHO Global Breast Cancer Initiative (GBCI) framework, we used a 90-day threshold to classify the overall care interval from symptom detection to treatment initiation (TTI). This reflects the GBCI’s recommended target of 60 days for diagnosis after symptom detection and 30 days from diagnosis to treatment [22]. We created a binary variable to categorize patients into, timely care (TTI ≤ 90 days) and delayed care (TTI > 90 days). The association between this binary TTI variable and FT scores was also evaluated using the Wilcoxon rank-sum test.

Multivariable linear regression was used to assess the independent association between TTI and FT scores, adjusting for variations of the covariates. All covariates (see details in the covariate section) were included simultaneously based on a priori conceptual relevance. Among those covariates, age, per-capita household income, family size, and distance to the first healthcare facility were initially recorded as continuous variables and subsequently categorised using clinically meaningful and context-relevant cut-offs, as no linear correlations between these variables and FT scores were assumed. Model assumptions were checked and multicollinearity among independent variables was assessed using the Variance Inflation Factor (VIF) [34] and all the VIF for the multivariate liner regression analysis were less than four. Results were reported as regression coefficients (*B*) with 95% confidence intervals (CI) and p-values. To assess the functional form of the association between TTI and FT, we constructed a shape plot of marginal means of the COST–FACIT score across TTI quintiles with 95% confidence intervals. TTI was divided into quintiles based on its empirical distribution. Adjusted COST–FACIT scores were estimated from a multivariable linear regression model including all prespecified covariates and plotted across TTI quintiles.

A sensitivity analysis using TTI as a continuous variable was conducted to test the robustness of the main model. All analyses were conducted using Stata version 19, and a p-value < 0.05 was considered statistically significant.

## Result

Among the 458 women with breast cancer included in this study, most were living in urban settings (74.0%) and were married (62.0%). Over half of the participants were recruited from Black Lion Hospital. The median age was 38 years (range: 22–77), and over half (51.8%) were in the 41–59-year age group. Slightly more than half were unemployed (51.5%) and 40% had a monthly household income of ≤ 2,100 ETB. The majority were covered by health insurance (80.1%), and 58.7% were diagnosed at stage III or IV. A prolonged interval (≥ 90 days) between symptom recognition and treatment initiation was reported by 325 (71.0%) patients. Additionally, 87 participants (19.0%) reported living with chronic conditions. To cope with OOP payments, 287 patients (62.7%) borrowed money from friends or other sources. Seventeen patients (3.7%) discontinued treatment due to cost: 6 skipped surgery, 9 chemotherapy, and 2 radiotherapy. Most patients received two treatment modalities (65.7%), were in the post-treatment stage (55.2%), and had ≤ 3 hospitalizations in the past year (96.5%). The median total treatment interval (TTI) was 181.5 days (IQR: 83–385 days), indicating substantial delays from symptom recognition to treatment initiation.

COST–FACIT scores were skewed toward lower values, indicating high financial toxicity among most participants (median 10, IQR 5–16). Financial toxicity was more pronounced among rural residents, unmarried women, those with lower educational attainment, and participants with lower per-capita household income. Patients with prolonged total treatment interval (> 90 days) had substantially high financial toxicity compared with those who initiated treatment within 90 days (9 vs. 14).

Patients diagnosed at Stage IV had high financial toxicity (the median COST–FACIT score 5). Higher financial toxicity was also observed among patients who relied on borrowed or assisted financing, used traditional healing, or avoided care due to cost. In contrast, patients receiving multiple treatment modalities reported comparatively lower financial toxicity. Financial toxicity was highest among patients who had just started treatment, who reported the lowest median COST–FACIT score (median 4.5), with scores progressively improving among those in later treatment stages and highest in the post-treatment group (Table 1).

**Table 1:** Distribution of COST-FACIT scores by participant characteristics

Characteristics	*n* (%)	Median (P_25_, P_75_)	*p*
**Age (Years)**			0.061
< 40	168 (36.7)	9 (5, 15)	
40–59	237 (51.8)	10 (6, 16)	
** ≥ **60	53 (11.5)	12 (6, 20)	
**Residence**			0.002
Urban	339 (74.0)	10 (6, 18)	
Rural	119 (26.0)	9 (4, 13)	
**Study site**			0.241
Black Lion	252 (55.0)	9.5 (5, 16)	
Hiwot Fana	65 (14.2)	9 (4, 18)	
Jimma	141 (30.8)	11 (7, 15)	
**Marital status**			0.023
Married	284 (62.0)	11 (6, 17)	
Unmarried	174 (38.0)	8.5 (5, 15)	
**Occupational status**			0.116
Employed	222 (48.5)	10 (6, 17)	
Unemployed	236 (51.5)	10 (4.5, 15.5)	
**Educational attainment**			< 0.001
No Formal Education	152 (33.1)	8 (4, 15)	
Primary and secondary education	208 (45.4)	10 (6, 17)	
Higher Education	98 (21.4)	12.5 (8, 18)	
**Monthly household income (ETB)**			< 0.001
≤ 2,100	183 (40.0)	7 (4, 13)	
2,101–3,254	161 (35.1)	10 (6, 15)	
3,255–15,000	114 (24.9)	14 (8.5, 22)	
**Distance to the health facility (km)**			0.060
< 5	350 (76.4)	10 (5, 17)	
≥ 5	108 (23.6)	10 (4, 13.5)	
**Traditional healing**			0.008
Yes	85 (18.6)	8 (4, 16)	
No	373 (81.4)	10 (6, 16)	
**Health insurance**			0.232
Yes	367 (80.1)	10 (5, 16)	
No	91 (19.9)	11 (6, 18)	
**TTI (days)**			< 0.001
≤ 90	133 (29.0)	14 (9, 21)	
> 90	325 (71.0)	9 (4, 14)	
**Family size**			0.234
< 2	96 (21.0)	8 (4.5, 16.5)	
2–5	297 (64.9)	11 (6, 16)	
> 6	65 (14.2)	9 (5, 14)	
**Stage at diagnosis**			< 0.001
Stage I	17 (3.7)	8 (4, 17)	
Stage II	172 (37.6)	11 (6.5, 17)	
Stage III	212 (46.2)	11 (6, 17)	
Stage IV	57 (12.5)	5 (3, 9)	
**Days since treatment initiation**			< 0.001
Just started	14 (3.1)	4.5 (3, 8)	
Early–mid treatment	105 (22.9)	8 (5,15)	
Late treatment	86 (18.8)	9 (4,14)	
Post-treatment	253 (55.2)	12 (6,18)	
**Medically confirmed chronic illness**			0.073
Yes	87 (19.0)	11 (7, 18)	
No	371 (81.0)	10 (5, 16)	
**Treatment modality**			< 0.001
Single modality	75 (16.4)	7 (4, 11)	
Two modaliti**es**	301 (65.7)	10 (5, 17)	
All three modality	82 (17.9)	13 (7, 18)	
**Hospitalizations (Past Year)**			0.376
0–3	442 (96.5)	10 (5,16)	
> 3	16 (3.5)	8 (4, 14.5)	
**Financial source of out-of-pocket money (OOP)**			< 0.001
Self-financed	167 (36.5)	13 (8–20)	
Borrowed/Assisted	291 (63.5)	9 (5–14)	
**Avoided care due to cost**			0.001
No	441 (96.3)	10 (5, 16)	
Yes	17 (3.7%)	3 (0, 10)	

Self-financed: Regular income, household saving, sold livestock and sold permanent asset; Borrowed/Assisted: borrowed from relatives/friends/money lenders, assistance/donation from relatives/friends

### Correlation between total treatment interval and financial hardship

**Fig. 1:**
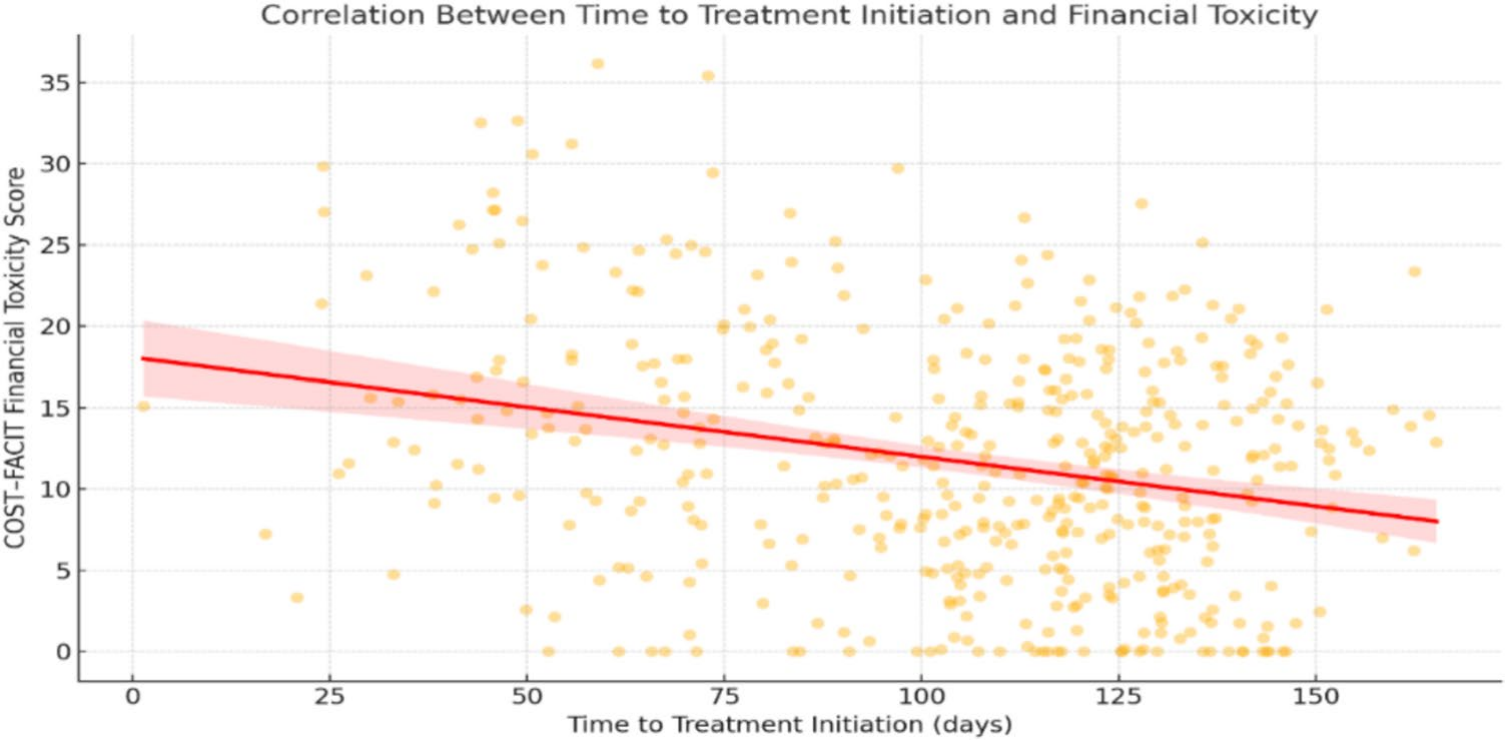
Correlation between TTI and COST–FACIT scores

Pearson’s correlation analysis demonstrated a significant negative linear association between COST-FACIT scores and TTI (r = –0.27, p < 0.001), suggesting that delays in treatment initiation were associated with greater financial toxicity (lower COST-FACIT scores) among breast cancer patients (Fig. 1).

### Multivariable modelling

In the fully adjusted model, prolonged total treatment interval (TTI > 90 days) was significantly associated with higher financial toxicity. Women who initiated treatment more than 90 days after symptom recognition had higher financial toxicity than those who initiated treatment within 90 days (B = –2.50; p = 0.002).

Age was associated with financial toxicity, with women aged 40–59 years and those under 40 years reporting higher financial toxicity compared to women aged ≥ 60 years. Patients in the initial–mid treatment phase also reported higher financial toxicity compared to those in the post-treatment phase. Lower per-capita household income and reliance on borrowed or assisted financing were associated with higher financial toxicity.

Geographical differences were observed, with rural residents and patients treated at Black Lion Hospital reporting higher financial toxicity. Other covariates were not significantly associated with COST–FACIT scores in the fully adjusted model (Table 2).

**Table 2:** Multivariable linear regression of factors associated with COST–FACIT scores

Variable	*B* Coefficient	95% CI	*p*	VIF
**Age (Years)**				
≥ 60	Ref			
40–59	−3.53	(−5.84, −1.23)	0.003	3.19
< 40	−4.37	(−6.90, −1.84)	0.001	3.57
**Marital status**				
Married	Ref			
Unmarried	−0.73	(−2.28, 0.81)	0.352	1.35
**Residence**				
Urban	Ref			
Rural	−2.28	(−4.44, −0.12)	0.039	2.16
**Study site**				
Jimma Hospital	Ref			
Black Lion Hospital	−2.79	(−4.71, −0.88)	0.004	2.18
Hiwot Fana Hospital	−1.12	(−3.39, 1.16)	0.335	1.51
**Monthly household income (Ethiopian Birr)**				
3,255–15,000	Ref			
2,101–3,254	−3.91	(−5.65, −2.17)	< 0.001	1.47
≤ 2,100	−5.40	(−7.19, −3.61)	< 0.001	1.82
**Educational attainment**				
Higher education	Ref			
Primary & Secondary education	1.56	(−0.37, 3.50)	0.114	2.23
No formal education	−0.01	(−2.46, 2.44)	0.994	3.20
**Occupational Status**				
Employed	Ref			
Unemployed	0.06	(−1.61, 1.73)	0.946	1.67
**Health Insurance**				
No	Ref			
Yes	1.30	(−0.40, 3.00)	0.133	1.11
**Medically Confirmed chronic illness**				
No	Ref			
Yes	1.01	(−0.77, 2.80)	0.265	1.12
**Days since treatment initiation**				
Post-treatment (> 365)	Ref			
Late treatment phase (181–365)	−1.62	(−3.47, 0.23)	0.087	1.25
Initial–Mid treatment phase (0–180)	−3.59	(−5.65, −1.53)	0.001	1.95
**Time to treatment interval**				
≤ 90 days	Ref			
> 90 days	−2.50	(−4.06, −0.95)	0.002	1.19
**Distance to the nearest health facility(km)**				
< 5 km	Ref			
≥ 5 km	−1.51	(−3.24, 0.22)	0.086	1.29
**Stage of cancer**				
Stage I & II	Ref			
Stage III–IV	0.34	(−1.18, 1.85)	0.663	1.33
**Hospitalizations (Past Year)**				
0–3	Ref			
> 3	0.62	(−3.05, 4.30)	0.740	1.09
**Treatment modality**				
Single	Ref			
Two	2.04	(−0.21, 4.31)	0.077	2.78
All three	0.50	(−2.33, 3.34)	0.727	2.83
**Traditional healing**				
No	Ref			
Yes	−0.08	(−1.86, 1.71)	0.931	1.15
**Family size**				
< 2	Ref			
2–5	1.40	(−0.41, 3.20)	0.129	1.77
> 6	2.28	(−0.42, 4.98)	0.097	2.12
**Financial source of ou** t**-of-pocket money (OOP)**				
Self-financed	Ref			
Borrowed/Assisted Financing	−4.59	(−6.04, −3.15)	< 0.001	1.16
**Avoided care due to cost**				
No	Ref			
Yes	−2.91	(−6.53, 0.71)	0.115	1.12

Final model: R^2^ = 0.331, adjusted R^2^ = 0.291, F (26, 431) = 8.20, *p* < 0.001. B, unstandardized regression coefficient

*VIF* Variance Inflation Factor

**Fig. 2:**
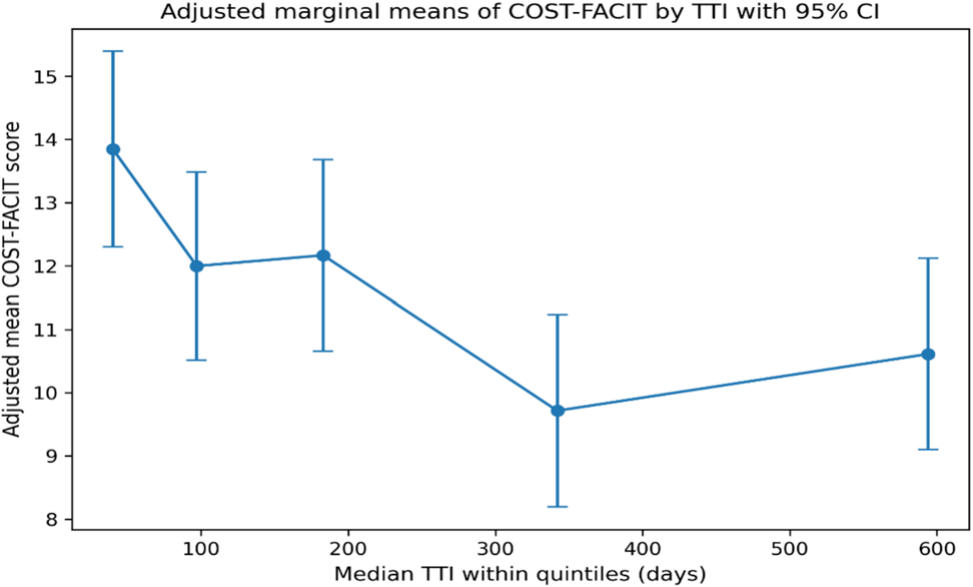
Adjusted marginal means of COST–FACIT scores plotted against the median time to TTI within each quintile. Vertical lines represent 95% confidence intervals from the multivariable model

The adjusted marginal means of COST–FACIT scores decreased with increasing time TTI, indicating greater financial toxicity with longer treatment delays (Fig. 2). Women in the shortest delay group (median TTI = 40 days) had the highest adjusted COST–FACIT scores (mean = 13.85; 95% CI: 12.30–15.39), reflecting lower financial toxicity. In contrast, women with longer delays had lower adjusted COST–FACIT scores, particularly in the fourth quintile (median TTI = 342 days; mean = 9.72; 95% CI: 8.20–11.23), indicating greater financial toxicity. Although there was a slight increase in the fifth quintile (median TTI = 594 days; mean = 10.61; 95% CI: 9.10–12.12), the overall pattern suggests worsening financial toxicity with longer TTI.

The association between COST–FACIT scores and TTI modelled as a continuous variable did not reach statistical significance (p = 0.134) in sensitivity analyses (Supplementary File 1).

## Discussion

The current study examined the association between timeliness of breast cancer care and financial toxicity measure by COST-FACIT, as well as other predictors of COST-FACIT scores. Women who initiated treatment more than 90 days after symptom detection, had a younger age, lived with lower household income, patients who relied on borrowed or assisted financing for OOP payments, resided in rural areas, being on active treatment, and received treatment in Black lion hospital reported higher financial toxicity. To our knowledge, the subjective experience of financial toxicity among breast cancer patients, and its association with delays in care, has not been previously studied in Ethiopia.

### The level of financial toxicity of patients with breast cancer in Ethiopia

Our study participants reported a median COST-FACIT score of 10 out of a maximum of 44. This extremely low score indicates that patient experienced pronounced financial toxicity. Similarly low COST-FACIT scores have been reported in other resource-limited settings. For example, a Nigerian study observed a median COST-FACIT score of 16 among women with cancer [35], and a Rwandan study of cancer patients during the COVID-19 lockdown found an average COST-FACIT score of only 9 [36]. However, the observed median COST-FACIT score in this study is substantially lower than those reported in middle- and high-income countries. For instance, median scores of 22 and 21, have been reported in the United State [37] and China [38] for breast cancer care, respectively.

The extreme financial toxicity observed in Ethiopian breast cancer patients is attributable to a combination of systemic health system factors and patient-level socioeconomic factors. In Ethiopia (as in many low-resource countries), there is little to no universal health coverage for cancer care [39]. The Ethiopian healthcare system provides very limited financial protection for expensive cancer treatments (e.g. chemotherapy, advanced surgeries). But household income is low, which often leads to catastrophic health expenditures. A study reported that 30% of Ethiopia’s health spending comes directly from patients [40]. Another review noted that 74.4% of Ethiopian cancer patients incur catastrophic healthcare costs (spending > 40% of their income on care) [39].

### Prolonged time to treatment and financial toxicity

Our study found that prolonged time from symptom recognition to treatment initiation is significantly associated with higher financial toxicity. The relationship between the two is likely to be bidirectional.

Delays in accessing care may contribute to financial toxicity through mechanisms beyond clinical severity alone. Studies from low-resource settings have shown that non-medical costs, particularly during extended diagnostic and pre-treatment intervals, can substantially increase the financial burden for cancer patients [41]. Prolonged delays often lead patients to make multiple trips, as they tend to present with more advanced or complex conditions requiring additional diagnostic tests, referrals, and follow-ups. These visits are usually to specialized urban hospitals, resulting in repeated travel and accommodation costs, particularly for treatments like radiotherapy, which require daily sessions over several weeks [42, 43], leading to rising logistical expenses that contribute to overall financial hardship [44]. Moreover, patients with prolonged delay often experience anxiety, depression, or other psychosocial distress while waiting for treatment [45], indirectly worsening financial toxicity by impairing a patient’s ability to manage work or personal finances [46].

On the other hand, financial toxicity may itself be a barrier to timely care, leading patients to avoid, postpone, or discontinue treatment due to lack of financial capacities. This is evident from many LMICs, where financial barriers are a key driver of care delays and often lead patients to postpone or skip recommended treatment [47, 48].

### Other factors associated with financial toxicity

Younger age was found to be associated with higher financial toxicity, consistent with previous studies [49, 50]. Notably, breast cancer in developing regions tends to occur at younger ages than in Western countries. In sub-Saharan Africa, for example, the average age at diagnosis is roughly 10 years younger than in Western nations [51]. Patients in their 30 s and 40 s are at a critical career stage, and cancer can disrupt their employment and earnings, intensifying financial strain. Younger women typically carry heavier financial burdens than older individuals, as they are often responsible for supporting dependents, paying bills, and covering day-to-day living expenses [52].

According to this study, financial toxicity is significantly associated with lower levels of monthly household income. This finding is consistent with other studies conducted elsewhere in the world, showing that patients with limited economic resources are more vulnerable to the financial impact of cancer care [8, 31, 38, 53, 54]. African governments spend under $100 per capita on health on average, versus several thousand dollars per capita in high-income countries [55]. In Ethiopia, direct patient payments make up the bulk of cancer care financing. A survey in Addis Ababa reported a mean total cost of cancer treatment of $2,366 per patient [6]. While our study focused on patients receiving care in public hospitals, it is important to note that the public oncology system in Ethiopia remains under-resourced. As a result, even patients treated in public facilities may be required to pay out-of-pocket for certain essential services, such as medications, diagnostic imaging, or timely surgeries, that are unavailable or delayed in the public sector. This partial reliance on private services can contribute substantially to financial hardship [56]. A survey of pharmacies in Addis Ababa found that the majority of essential cancer drugs were not available in the public sector, and those that were available privately were unaffordable to most patients [57]. This scarcity in the public supply chain pushes patients to buy high-priced drugs out-of-pocket or go without treatment. Besides, our study found that breast cancer patients who relied on borrowed or assisted financing reported significantly greater financial toxicity than those who self-finance their care, a finding that aligns with previous studies [38, 58]. Financial insecurity can also increase material and psychological stress, worsening financial toxicity [59].

This study showed that patients in the initial–mid treatment phase had significantly greater financial toxicity than those in the post-treatment phase. Previous studies reported that patients who were receiving active treatment were exposed to greater financial toxicity than those whose treatment had completed [60, 61]. This may be explained by the nature of the COST-FACIT tool, which captures patients perceived financial burden, often most acute during active treatment when OOP expenses for medications, travel, diagnostics, and income loss are highest [30]. Over time, patients may adapt to these financial demands, encounter fewer direct costs, or experience psychological relief after completing treatment, leading to a more favourable perception of their financial situation.

Geographic disparities in financial toxicity exist in Ethiopia, according to the findings of this study. Patients residing in rural areas experience higher levels of financial toxicity, a pattern consistently supported by previous research [62, 63]. Rural patients usually have low health-insurance literacy, contributing to inability to use insurance effectively and appropriately. Rural residency also imposes increased travel and lodging costs, greater loss of income, and significant access barriers for cancer care in specialised hospitals usually in urban locations [62, 64]. Moreover, this study found that patients receiving care at Black Lion Hospital experienced significantly higher financial toxicity. This may be attributed to the hospital’s location in the capital city and its role as a national referral centre, particularly for specialised treatments such as radiotherapy, which are scarcely available elsewhere in Ethiopia. As a result, patients referred to Black Lion often incur substantial travel and accommodation costs (since therapy like radiotherapy requires multiple sessions over weeks), along with prolonged waiting times, further exacerbating their financial burden [65].

In contrast to several prior studies that identified advanced cancer stage as a significant predictor of financial toxicity [27, 38, 44], our study found no statistically significant association between stage at diagnosis and COST-FACIT scores in the multivariable analysis. Although patients diagnosed with stage IV disease reported higher levels of financial toxicity in the univariate analysis, this association did not persist after adjusting for other factors. One possible explanation is that in low-resource settings like Ethiopia, financial burden may be high across all cancer stages due to widespread OOP payments, limited financial protection, and high indirect costs (e.g., travel, accommodation, and income loss). Similarly, while the univariate analysis suggested that patients receiving a single treatment modality experienced greater financial toxicity compared to those receiving two or three modalities, this association also lost significance in the multivariable model. This may reflect socioeconomic differences in treatment access, where patients who can afford multiple treatment modalities are also better positioned to manage the financial burden of care.

In this study, health insurance was not statistically significantly associated with financial toxicity, suggesting that existing health insurance schemes in Ethiopia may not provide effective financial protection for cancer patients. By contrast, patients in middle- and high-income countries often have insurance (public or private) that covers a significant portion of treatment costs, reducing direct OOP.

### Policy recommendations

These findings indicate that multiple policies or actions should be taken by the government and society to relieve the financial difficulties faced by patients with breast cancer, especially those who experience prolonged delay, are younger, live with low household income, and reside in rural area. Although Ethiopia has made progress toward universal health coverage through initiatives such as community-based health insurance (CBHI), current financial protection mechanisms remain insufficient to adequately shield cancer patients from the economic burden of care. Key actionable recommendations include: (1) strengthening financial risk protection for cancer care through expanded insurance coverage; (2) prioritising early diagnosis and streamlined referral pathways to reduce both clinical and financial burden; (3) introducing targeted social support mechanisms to offset indirect costs such as transport and accommodation; and (4) integrating routine financial toxicity screening into oncology care to enable early identification and support of high-risk patients.

## Strengths and limitations

Our study was conducted across three major tertiary hospitals in Ethiopia, enhancing the representativeness and generalizability of the findings within the national context. This is the first study to translate, culturally adapt, and validate the COST–FACIT tool into Amharic, ensuring linguistic and contextual relevance for Ethiopian cancer patients. The research addresses a major knowledge gap by exploring the relationship between treatment delays and financial hardship in a low-resource setting, contributing valuable context-specific evidence to the global literature. However, this study has some limitations. First, the nature of cross-sectional design limited the power to determine a causal relationship. Second, the primary exposure variable, TTI, relied partly on patients' recall of symptom onset, which may be prone to recall bias. Thirdly, although patients were recruited at various stages of treatment, the study design does not allow for tracking the entire journey of cancer patients over time. And treatment stage was inferred based on time since treatment initiation, which may not perfectly reflect actual treatment completion or survivorship status, particularly in low-resource settings where treatment interruptions may occur. Fourthly, patients who discontinued treatment or never reached tertiary cancer centres were not captured in this study, leading to a possible underestimation of the true impact. Although the study included three major tertiary centres, participants were recruited from facility-based settings using a consecutive sampling approach. As a result, the findings may not be fully generalisable to all women with breast cancer in Ethiopia, particularly those managed in primary or secondary facilities, rural settings, or those unable to access specialised cancer care. Finally, while the COST–FACIT instrument is a validated measure of patient-reported financial toxicity, it has limitations in low- and middle-income settings. Developed in the United States, it primarily captures individual-level financial distress and may not fully reflect the generational and household-level financial impacts common in contexts such as Ethiopia, where cancer costs are often absorbed through family borrowing, asset sales, and long-term economic strain.

## Conclusion

This study highlights a high level of financial toxicity among Ethiopian women with breast cancer. Prolonged TTI, younger age, low income, rural residency, relying on borrowed or assisted financing, being on active treatment, and received treatment in Black lion hospital are significant predictors of higher financial hardship. However, health insurance appears to be insufficient to alleviate financial hardship. These findings underscore the urgent need for health system reforms to reduce delays in care, decentralise cancer services, and expand effective financial protection mechanisms. Targeted policy interventions to subsidise cancer treatment costs, improve access to essential medicines in the public sector, and address socioeconomic-related inequalities are needed for reducing the burden of breast cancer in low-resource settings like Ethiopia. At the same time, further research is needed to guide these efforts, including longitudinal studies to better establish the temporal and causal relationships between treatment delays and financial toxicity, community-based studies that capture women who never reach tertiary cancer centres, and intervention studies to evaluate health system, financial protection, and social support strategies aimed at reducing financial toxicity.

## Supplementary Information

Below is the link to the electronic supplementary material.

Supplementary file1 (DOCX 21 KB)

## Data Availability

The datasets generated and/or analysed during this study are not publicly available due to the inclusion of potentially identifiable health information. Data may be made available upon reasonable request, subject to approval by the La Trobe University Human Research Ethics Committee. Interested researchers should contact Professor George Liu ([C.Liu@latrobe.edu.au](mailto:C.Liu@latrobe.edu.au)) or the Ethics Committee ([humanethics@latrobe.edu.au](mailto:humanethics@latrobe.edu.au)).

## Abbreviations

*NCCP*: National Cancer Control Plan
*OOP*: Out-of-pocket
*COST–FACIT*: Comprehensive Score for Financial Toxicity – Functional Assessment of Chronic Illness Therapy
