# Supplementary material for: Association between time to treatment and financial toxicity among women with breast cancer in Ethiopia: a multicentre study in a low-resource setting
Source: Support Care Cancer. 2026 Mar 13;34(4):314. doi: 10.1007/s00520-026-10570-9 (PMC12987774; doi:10.1007/s00520-026-10570-9)
Supplement: Supplementary file 1 — Supplementary file1 (DOCX 21 KB) [file 520_2026_10570_MOESM1_ESM.docx]

**Supplementary file table 1: Multivariable linear regression of factors associated with financial toxicity (COST–FACIT Score): Continuous TTI Model**

| Variable | *B*(Coefficient) | 95% CI | p | VIF |
| --- | --- | --- | --- | --- |
| Time to Treatment Interval (TTI) | -0.0018 | (-0.0041, 0.0005) | 0.134 | 1.21 |
| Age in years |  |  |  |  |
| >=60 | Ref. |  |  |  |
| 40–59 | -3.62 | (-5.95, -1.29) | 0.002 | 3.20 |
| <40 | -4.34 | (-6.90, -1.78) | 0.001 | 3.59 |
| Monthly household Income (ETB) |  |  |  |  |
| 3,255–15,000 | Ref. |  |  |  |
| 2,101–3,254 | -3.94 | (-5.69, -2.18) | 0.000 | 1.47 |
| <=2,100 | -5.62 | (-7.42, -3.82) | 0.000 | 1.81 |
| Educational attainment |  |  |  |  |
| Higher education | Ref. |  |  |  |
| Primary & Secondary | 1.59 | (-0.36, 3.54) | 0.111 | 2.23 |
| No formal education | -0.11 | (-2.59, 2.36) | 0.928 | 3.20 |
| Occupational status |  |  |  |  |
| Employed | Ref. |  |  |  |
| Unemployed | 0.10 | (-1.58, 1.79) | 0.905 | 1.67 |
| Health insurance |  |  |  |  |
| No | Ref. |  |  |  |
| Yes | 1.35 | (-0.37, 3.06) | 0.124 | 1.11 |
| Medically confirmed chronic illness |  |  |  |  |
| No | Ref. |  |  |  |
| Yes | 0.98 | (-0.83, 2.78) | 0.288 | 1.18 |
| Distance to nearest health facility |  |  |  |  |
| <5km | Ref. |  |  |  |
| >=5km | -1.51 | (-3.26, 0.23) | 0.089 | 1.29 |
| Stage of cancer |  |  |  |  |
| Stage I & II | Ref. |  |  |  |
| Stage III–IV | 0.11 | (-1.40, 1.63) | 0.882 | 1.32 |
| Marital status |  |  |  |  |
| Married | Ref. |  |  |  |
| Unmarried | -0.75 | (-2.31, 0.81) | 0.345 | 1.36 |
| Residence |  |  |  |  |
| Urban | Ref. |  |  |  |
| Rural | -2.52 | (-4.70, -0.34) | 0.023 | 2.15 |
| Study site |  |  |  |  |
| Jimma | Ref. |  |  |  |
| Black Lion | -3.05 | (-4.98, -1.12) | 0.002 | 2.18 |
| Hiwot Fana | -0.98 | (-3.27, 1.32) | 0.405 | 1.52 |
| Traditional healing |  |  |  |  |
| No | Ref. |  |  |  |
| Yes | -0.15 | (-1.97, 1.66) | 0.867 | 1.18 |
| Treatment modality |  |  |  |  |
| Single | Ref. |  |  |  |
| Two | 1.98 | (-0.34, 4.30) | 0.094 | 2.86 |
| All three | 0.56 | (-2.33, 3.44) | 0.705 | 2.89 |
| Days since treatment initiation |  |  |  |  |
| Post-treatment (>365) | Ref. |  |  |  |
| Late phase (181-365) | -1.61 | (-3.48, 0.26) | 0.091 | 1.26 |
| Early–Mid phase (0-180 days) | -3.65 | (-5.72, -1.57) | 0.001 | 1.95 |
| Family size |  |  |  |  |
| <2 | Ref. |  |  |  |
| 2–5 | 1.22 | (-0.60, 3.03) | 0.188 | 1.77 |
| >6 | 1.78 | (-0.93, 4.50) | 0.198 | 2.12 |
| Avoided care due to cost |  |  |  |  |
| No | Ref. |  |  |  |
| Yes | -3.17 | (-6.82, 0.48) | 0.089 | 1.12 |
| Financial source of out-of-pocket money (OOP) |  |  |  |  |
| Self-financed | Ref. |  |  |  |
| Borrowed/Assisted | -4.78 | (-6.23, -3.33) | 0.000 | 1.15 |
| Hospitalization |  |  |  |  |
| 0–3 | Ref. |  |  |  |
| 4–7 | 0.48 | (-3.24, 4.20) | 0.799 | 1.10 |
